# Preoperative Echocardiographic Unknown Valvopathy Evaluation in Elderly Patients Undergoing Neuraxial Anesthesia during Major Orthopedic Surgery: A Mono-Centric Retrospective Study

**DOI:** 10.3390/jcm13123511

**Published:** 2024-06-15

**Authors:** Antonio Coviello, Dario Cirillo, Maria Vargas, Andrea Uriel de Siena, Maria Silvia Barone, Francesco Esposito, Antonio Izzo, Pasquale Buonanno, Serena Volpe, Andrea Gabriele Stingone, Carmine Iacovazzo

**Affiliations:** 1 Department of Neurosciences, Reproductive and Odontostomatological Sciences, “Federico II”—University of Naples, 80100 Naples, Italy; antonio_coviello@live.it (A.C.); vargas.maria82@gmail.com (M.V.); andreauriel@outlook.it (A.U.d.S.); baronemariasilvia@gmail.com (M.S.B.); dr.esposito@hotmail.com (F.E.); pasquale.buonanno@unina.it (P.B.); svolpe204@gmail.com (S.V.); stingoneandrea@gmail.com (A.G.S.); dott.iacovazzo@gmail.com (C.I.); 2 Unit of Orthopedics and Traumatology, Department of Public Health, School of Medicine, “Federico II”—University of Naples, 80100 Naples, Italy; izzoantonio1992@gmail.com

**Keywords:** regional anesthesia, preoperative cardiac risk assessment, orthopedic revision surgery, cardiac risk scores, preoperative echocardiography screening

## Abstract

**Background:** The assessment of cardiac risk is challenging for elderly patients undergoing major orthopedic surgery with preoperative functional limitations. Currently, no specific cardiac risk scores are available for these critical patients. Echocardiography may be a reliable and safe instrument for assessing cardiac risks in this population. This study aims to evaluate the potential benefits of echocardiography in elderly orthopedic patients, its impact on anesthesiologic management, and postoperative Major Adverse Cardiac Events (MACEs). **Methods:** This is a retrospective, one-arm, monocentric study conducted at ‘’Federico II’’ Hospital—University of Naples—from January to December 2023, where 59 patients undergoing hip or knee revision surgery under neuraxial anesthesia were selected. The demographic data, the clinical history, and the results of preoperative Echocardiography screening (pEco-s) were collected. After extensive descriptive statistics, the χ^2^ test was used to compare the valvopathies and impaired Left Ventricular Function (iLVEF) prevalence before and after echocardiography screening and the incidence of postoperative MACE; a *p*-value < 0.05 was considered statistically significant. **Results:** The mean age was 72.5 ± 6.9, and the prevalence of cardiac risk factors was about 90%. The cumulative prevalence of iLVEF and valvopathy was higher after the screening (*p* < 0.001). The pEco-s diagnosed 25 new valvopathies: three of them were moderate–severe. No patients had MACE. **Conclusions:** pEco-s evaluation could discover unknown heart valve pathology; more studies are needed to understand if pEco-s could affect the anesthetic management of patients with functional limitations, preventing the incidence of MACE, and assessing its cost-effectiveness.

## 1. Introduction

Preoperative cardiology risk assessment should be based on a clinical evaluation and many scores, including Metabolic Equivalent Task score (METs), Revised Cardiac Risk Index (RCRI), American College of Surgeons—National Surgical Quality Improvement Program (ACS-NSQIP), Surgical Outcome Risk Tool (SORT), Surgical Risk Calculator, American University of Beirut Cardiovascular Risk Index (AUB)-HAS2, and the Duke Activity Status Index (DASI) [1,2,3,4,5,6,7]. However, none of these scores are effective in assessing cardiological risk and preventing postoperative Major Adverse Cardiac Events (MACEs). The clinician ultimately makes the decision [8,9]. Assessing patients solely based on clinical scores can be inadequate and incomplete, especially for patients with functional limitations [10]. Additional cardiological investigations, such as echocardiography, may be useful in diagnosing potentially life-threatening heart disease, even in patients with known or unknown cardiac risk factors [11]. Despite advances in diagnostics and medical research, heart valve diseases still represent a growing cause of global cardiovascular morbidity and mortality; aortic stenosis is the most common condition in developed countries [12]. In developing countries, limited access to echocardiography probably leads to a large underestimation of the number of people with valvular disease [13]. Recent studies found that about 6.4% of the general population have undiagnosed valvopathies, which could be moderate or severe heart valve abnormalities; the estimated incidence of aortic stenosis is about 1.3% among the population affected by valvopathy [14].

Ultrasound scans have revealed that 39% of patients undergoing hip fracture surgery have aortic stenosis; among these, the prevalence of moderate or severe disease is about 8% [15]. Even if some biomarkers increased in heart disease, such as B-natriuretic peptide in Heart Failure (HF) or aortic mixed valve disease, their serum level is not well correlated with the risk of MACEs [16,17]. Heart valve diseases impose an additional burden on the cardiovascular system and increase the MACEs during and after surgery, such as Myocardial Infarction (MI), arrhythmias, HF, stroke, and death, even in the absence of significant hemodynamic changes [18]. The incidence of MI or congestive HF seems to be about 1–2% in elderly patients undergoing major orthopedic surgery; total hip arthroplasty reported the highest odds ratio (4.17) compared to other orthopedic surgeries [19]. Aortic stenosis negatively influences postoperative outcomes in patients undergoing Non-Cardiac Surgery (NCS), such as 30-day mortality and postoperative MI [20]. Therefore, the mortality of elderly patients undergoing major orthopedic surgery is very high if MI occurs (13.9% in patients with MI vs. 3.2% in patients without MI) [21]. MI has a heavy economic impact on elderly patients: an economic analysis conducted in the USA showed that in-hospital costs average USD 18,931 and 1-year costs average USD 8037 [22]. However, before the evaluation of the economic cost-effectiveness of the diagnostic and risk assessment tools in preventing MACEs in elderly patients undergoing major orthopedic surgery, how these tools impact clinical management must be analyzed. Early recognition and appropriate management of undiagnosed heart valve diseases are crucial for improving clinical outcomes and patient quality of life and reducing the occurrence and economic impact of MACEs. Our study aims to evaluate whether echocardiography should be performed on a specific group of patients who are not typically assessed with this diagnostic procedure according to current guidelines. We hypothesize that, in patients affected by functional limitations, preoperative Echocardiography screening (pEco-s) may impact anesthesiologic management and, by targeting the anesthesia also on echocardiography findings, may lower the incidence of MACEs, such as Myocardial Ischemia (MI), ICU admission, and death. Through a retrospective assessment, we aim to quantify the unidentified valvopathies and determine how many of them, thanks to echocardiography screening and targeted anesthesiologic management, did not undergo MACEs.

## 2. Materials and Methods

The study was conducted in accordance with the Declaration of Helsinki or equivalent ethical standards. Institutional ethical committee approval was not necessary since the data were collected during daily clinical practice. All patients who provided informed consent for anesthesia also agreed to the use of their data anonymously for scientific purposes. The study was conducted according to the Strengthening the Reporting of Observational Studies in Epidemiology (STROBE) statement [23].

### 2.1. Study Design and Study Population

This study was a Level III monocentric retrospective study performed at the “Department of Surgical Sciences, Orthopedic Trauma, and Emergencies’’ of “Federico II”—University of Naples (Naples, Italy). The study focused on patients who underwent elective orthopedic hip and knee revision surgery under neuraxial anesthesia at our institution from January 2023 to December 2023. The data were retrieved from the department archive, recorded on a pre-filled form, and stored in a password-protected computerized database using Microsoft Office Excel 2016 (Microsoft, Redmond, WA, USA) in a secured computer of the department. The inclusion criteria were patients with functional limitations aged more than 65 years old scheduled for elective orthopedic hip and knee revision surgery due to septic or non-septic cause; a Body *Mass* Index (BMI) of 18–45 kg/m^2^ based on the weight the day before surgery and the height measured at admission to the hospital, divided into three classes (the first 30–35, the second 35–40, the third 40–45); an American Society of Anesthesiologists (ASA) physical status classification between I and IV; the presence of pEco-s data, performed a maximum of 3 months before surgery; the use of neuraxial anesthesia as anesthesiologic intraoperative approach (epidural, subarachnoid, or continuous spinal anesthesia (CSA). The pEco-s was conducted according to a local protocol used in our hospital throughout 2023, which established multiple screenings for elderly patients undergoing major or intermediate–major surgery. The pEco-s was conducted with the Philips iE33 machine. According to the same local protocol, all patients had to be assessed with appropriate scales for functional evaluation and with cardiac risk scores.

### 2.2. Functional Evaluation and Cardiac Risk Assessment

The enrolled patients developed a functional impairment in daily and living functionality due to the orthopedic disease: the patient affected by hip or knee prosthesis infection or dislocation/rupture cannot practice sports and, in some cases such as Hofmann’s spacer, cannot move the limb [24,25]. The evaluation of functional impairment was assessed with appropriate scores for sports and daily functions: the Knee Osteoarthritis Outcome Score Injury (KOOS-I) and Hip Osteoarthritis Outcome Score (HOOS) were used to assess the impairment for patients undergoing knee or hip surgery revision, respectively [26,27]. A patient was classified ‘functionally limited’ by the orthopedic disease if the KOOS-I or HOOS sport subscale was less than 15% and the KOOS-I daily or HOOS living subscale was less than 25%; all scores were administered in the official validated Italian version [26,27].

The preoperative cardiac risk assessment was conducted with the following tools: pre-pathology METs and preoperative MET, RCRI, and ACS-NSQIP. The scores ranked the patients in the following way: METs less than 4 defined a high cardiac risk; the RCRI was divided into four classes according to the total score between 0 and 4 (class I-3.5% of risk and 0 points; class II-6% of risks and 1 point, class III-10.1% and 2 points, and class IV-15% of risk and 4 points); and the ACS-NSQIP was considered at high risk if the cumulative risk was greater than 6 events per 1000 patients, compared with the same conditions for the same type of surgery [1,2,3]. Cardiovascular risk factors were considered and collected according to the 2022 ESC guideline [8,9]. The exclusion criteria were the following: incomplete clinical data recorded, absence of the preoperative cardiac risk assessment, and psychiatric or central degenerative conditions.

### 2.3. Data Extraction

The individual data of each patient were manually extracted from the clinical records. The data extracted were the following: the demographic and anthropometric data of the patients, such as age, weight, height, BMI, and sex; type of surgery (hip and knee revision surgery); basal hemoglobin levels; preoperative blood pressure, evaluated before the execution of neuraxial anesthesia; and KOOS-I and HOOS scores [26,27]. Additionally, the records cover the ASA physical status classification, medical history, and risk factors such as obesity, smoking, and cardiovascular disease. Furthermore, the records include MET scores previous to and after the acute orthopedic disease, preoperative RCRI score, and preoperative ACS-NSQIP cardiac event risk scores. The data also reported information about left ventricular ejection fraction (LVEF); if the estimated LVEF with modified Simpson’s biplane method was less than 50%, the LVEF was considered impaired (ILVEF) [28,29]. In addition, the records contain details about the number of patients with valvopathy and its features, evaluated by the cardiologist according to the 2021 and 2022 ESC/ESAIC Guidelines and its experience, the type of neuraxial anesthesia performed, and the MACE after surgery (symptomatic MI, stable or unstable angina, death) [8,9,28,29].

### 2.4. Outcomes

The main outcomes were the difference between the number of diagnosed diseases and the number of known diseases in patients undergoing elective hip or knee surgery and the incidence of MACEs (symptomatic MI, stable or unstable angina, death).

### 2.5. Statistical Analysis

The total sample size was calculated considering an effect size W of 0.4 based on the difference between the categorial distribution of iLVEF and valvulopathies before and after the pEco-s, an α error of 0.05, a β error of 0.2, a power (1- β) of 0.8, and freedom degrees equal to 1 using G* power v3.1.9 [30]. The total sample size was 50, with a critical χ^2^ of 3.84. Parametric data were presented as mean and standard deviation (SD); non-parametric data were reported as median and interquartile range (IQR). All continuous data were approximated to the first decimal place. The analysis of continuous data was conducted with the student *t*-test for parametric data and the Mann–Whitney U-test for non-parametric data. The dichotomous data were reported as the absolute number of observations and relative frequencies. Descriptive statistics were used to describe the prevalence of risk factors, the characteristics of the sample, the number of known and newly diagnosed valvopathies, the categorical distribution of the scores, and the incidence of MACE. A χ^2^ was used to analyze the dichotomous distributions. All the statistical tests were considered significant if the *p*-value was less than 0.05. After the overall analysis, the newly diagnosed valvopathies were divided into four different grades (mild, moderate, moderate–severe, and severe); their absolute and relative frequencies are presented. All the analyses were performed using R v4.0.0 and its basic package ‘stats’ [31].

## 3. Results

### 3.1. Screening and Demographic Data

**Figure 1:**
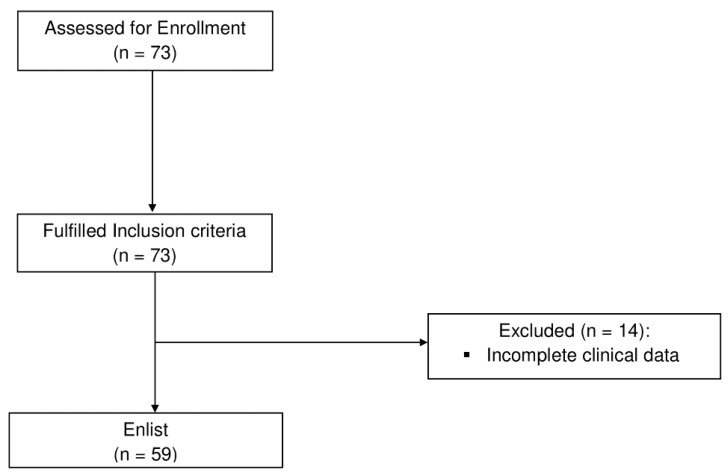
Flowchart of the study.

After screening 73 clinical records, 14 clinical records were excluded due to lack of data; finally, the data of a total sample size of 59 patients were recorded (see Figure 1).

The sample size is composed of 45 (74.6%) females and 16 (25.4%) males with a mean age of 72.5 ± 6.9 and a mean BMI of 30.5 ± 5.1 (see Table 1). Among the 59 patients treated for joint revision surgery, 29 were treated for hip and 30 for knee (49.1% vs. 50.1%). The baseline functional status assessed with KOOS-I and HOOS tools reported a score of 15.6 ± 6.0 and 16.2 ± 5.1 for the daily function domain, respectively; moreover, the KOOS-I sports domain score was 8.3 ± 4.7. Meanwhile, the HOOS sports domain scored 8.1 ± 5.1. The ASA physical status classes were distributed as follows: 3 patients were rated as class I; 35 patients were evaluated as class II; 17 patients were assessed as class III; and 4 patients as class IV.

### 3.2. Cardiac Risk Factors

#### 3.2.1. Cardiac Risk Factors Distribution

The analysis of cardiac risk factors showed a prevalence of 45.9% for obesity, with the first class more represented (67.9% vs. 21.4% vs. 10.7%, *p* < 0.001) and a prevalence of only 12 smokers (19.7%). Moreover, 55 patients were affected by cardiovascular disease (see Table 2). Among cardiovascular diseases, hypertension (83.61%) was the most frequent cardiovascular pathology, followed by valvopathy disease (47.3%) (see Table 2). The analysis of other diseases showed that chronic obstructive pulmonary disease (COPD) and diabetes were the third and fourth most prevalent diseases (32.8% and 20.3%, respectively) (see Table 2).

#### 3.2.2. Scores for Cardiac Factors Assessment Results

The preoperative cardiac risk evaluation via tools resulted in a high-risk score for MACEs in most observations (see Table 3). The analysis of METs showed the patients before and after the recrudescence of the orthopedic disease with high risk (METs < 4) were 7 and 54, respectively (11.9% vs. 91.5%, *p* < 0.001) (see Table 3). The RCRI categorization reported that 41 patients were evaluated as class I risk, 17 patients as class II, and only 1 as class III (see Table 3). The ACS-NSQIP evaluation reported that 29 patients undergoing hip revision surgery (100%) and 24 patients undergoing knee revision surgery (80.0%) have a risk greater than 0.006% for MACE, with no statistically significant difference between the patients undergoing hip or knee revision surgery (see Table 3).

### 3.3. Echocardiography Evaluation

#### 3.3.1. Comparison between Pre and Post Echocardiography Screening

The cumulative prevalence of iLVEF and valvopathies was higher after echocardiography screening (45.9% vs. 77.0%, *p* < 0.001) (see Table 4). In addition to the 11 patients with known valve disease, 12 patients had a diagnosis of a valve disease by the echocardiography screening (18.6% vs. 45.1%, *p* = 0.003). In the stratified analysis, no statistically significant difference was found between the known and newly diagnosed iLVEF; only the valvopathies were significantly higher after the screening (27 vs. 43, *p* < 0.001) (see Table 4). However, no statistically significant differences were found between the distributions of each type of valve disease.

#### 3.3.2. New Valve Disease Classification

The total number of newly diagnosed valve diseases was 25 (see Table 5). The analysis of the new 25 valve diseases was divided as follows: 21 were mild insufficient valves (seven aortic valves, six tricuspid valves, eight mitral valves), one was a moderate insufficient aortic valve, one was a moderate–severe insufficient tricuspid valve, and two were severe aortic stenosis (see Table 5). The anesthesiologist performed 0 epidural catheter placement, 56 subarachnoid single-shot anesthesia, and 3 CSAs (0.0% vs. 95.0% vs. 5.0%, *p* = 0.002); all the CSAs were performed only in patients affected by moderate–severe or severe valve disease discovered by the pEco-s. No MI, stable and unstable angina, ICU admission, or death were reported.

## 4. Discussion

Our population was composed of elderly elective surgical patients undergoing hip or knee elective surgery revision with a heavy impairment of the functional status due to the orthopedic pathology, assessed with appropriate scores, and screened with pEco-s according to our internal protocol. The prevalence of cardiovascular risk factors was high; hypertension resulted in the most diffused cardiovascular pathology, and first-class obesity was the most prevalent comorbidity. The assessment of cardiac risk with METs and ACS-NSQIP scores revealed that most patients had a low cardiac functional reserve and a higher risk of MACEs than average; meanwhile, the RCRI classified the risk of MACEs between 3.5% and 10.1% in more than half of patients. The pEco-s revealed 25 new valvopathies, which were previously unknown, in 12 patients. Among these, three of them suffered from moderate–severe tricuspid insufficiency or severe aortic stenosis pathology; all the other patients had mild to moderate valvopathy. The diagnosis of these three pathologies with a high risk of complications leads the anesthesiologist to the use of CSA to reduce the risk of hypotension and cardiac stress. No patients had MACEs (MI or stable and unstable angina) or were admitted to the ICU, probably due to the pEco-s that led to targeted anesthesiologic management. Unfortunately, the cost data for the echocardiography approach are missing; moreover, to assess the cost-effectiveness of this type of screening in this population, the cost of the MACE should be considered not only in the hospital setting but also in the long-life term, both social and health costs, as occurred for sugammadex [32].

Current guidelines provide several available risk scores to predict the likelihood of cardiovascular events during and after NCS [8,9]. However, these risk scores have certain limitations and may not be accurate for elderly orthopedic patients with functional limitations [8,9]. According to our analysis, the cumulative cardiac risk for MACEs assessed with different scores was often high: MET score was often less than 4, ACS-NSQIP reported a risk of 6 events per 1000 patients in most patients, and RCRI scored the risk between 3.5% and 10.1%. However, only RCRI seemed to rank the risk of these elderly orthopedic patients.

A review conducted by Franklin et al. raised concerns about METs; it concludes that the patient’s capacity for physical activity is often overestimated [33]. In addition, Tompuri et al. stated that the use of standard METs, which are based on body weight, can be altered by adiposity and suggested using lean mass proportional measures for a more accurate assessment of physical activity [34]. Interestingly, the application of METs in the studied patients seems to be impaired by the underlying orthopedic pathology; indeed, the lowering of functional status due to the impossibility of movement reduced the MET score even if there was no worsening in the patient’s cardiac function. Indeed, the use of METs is not suggested by the current guidelines in impaired functional patients [8,9]. The application of METs seems to not be tailored to a population affected by functional impairment only due to orthopedic conditions; more studies are needed to understand the efficacy of METs in assessing the functional reserve in patients undergoing knee or hip surgery revision with a temporary function impairment.

Davis and colleagues found that the RCRI may not be accurate in predicting complications in certain patient populations, in particular, the elderly and those with poor general health status [35]. Similarly, Andersson et al. noted that the performance of the RCRI varies between different age groups [36]. According to our results, the application of RCRI seems to be stratified better by orthopedic elderly patients than METs due to the possibility of assessing risk according to the patient’s history and not only on the supposed cardiac functional reserve. Based on a recent meta-analysis, the RCRI score seems to be the best for assessing the risk of MACEs in elderly patients undergoing NCS [2]. However, a meta-analysis enrolling patients undergoing major orthopedic surgery suggested that the RCRI score is not well correlated with the risk of MACE [37]. Other than the RCRI score, ACS-NSQIP is also used for preoperative cardiac risk assessment of MACEs [2,5,38]. The ACS-NSQIP cited by ACC/AHA 2014 guidelines reported the evaluation of functional capacity. The application of these scores on the evaluated elderly orthopedic surgery undergoing hip or knee revision is not possible due to the temporary constriction of the patients in bed; moreover, the ACS-NSQIP could be tailored to the surgery [38,39] or, as occurred for preoperative anxiety [40], a validated score for this type of patients should be established.

Molina and colleagues also conducted a study to evaluate the ACS-NSQIP in patients undergoing orthopedic surgery [41]. They concluded that the ACS-NSQIP had limitations in collecting data only up to the 30th postoperative day, which resulted in an underestimation of major complications that may occur beyond the 30th day, such as cardiopulmonary complications [41]. New diagnostic tools are required because of the reliability uncertainty of specific scores. The ACS-NSQIP had the same results as the METs score in our population. The risk of cardiac events was estimated as high with no difference in surgery, even if not all the patients had the same risk. All three assessed scores were not specific and reliable in patients with low function, primarily due to an orthopedic condition related to the hip or knee. It is also important to underline that the Perioperative Management of Elderly (PriME) Italian guidelines suggested the use of ACS-NSQIP for the assessment of MACE risk in NCS [42]. Echocardiography could be a valuable option for low-functional elderly patients, as it can detect valvular pathologies and other unknown pathologies that are more common in elderly age groups [43,44]. Indeed, after encountering several complications during and after surgery, such as bradycardia, hypotension, and acute coronary syndrome in patients with undiagnosed valvular disease and where the MET score failed to screen a high-risk, the first of which was in 2020, it was decided to perform echocardiography on all patients with functional limitations with the evaluation of other scores such as METs, like RCRI and ACS-NSQIP [45].

The pEco-s revealed new valvular diseases—one of them was a moderate–severe insufficient tricuspid, and two of them were aortic stenosis; this finding led to a change in anesthesia by using CSA. Van Heur et al. stated that valve disease and left ventricular systolic dysfunction are the two most common abnormalities identified by echocardiography [46]. The structural disease may not be suspected from the clinical request, as shown by Chambers et al.’s study in which the use of echocardiography was limited only in the presence of a murmur of probable valve disease. The estimated prevalence of valve disease was about 7% in the population of the study, unlike our study, in which the prevalence of known valve diseases was 18.6% [46]. Extending the use of echocardiography to the entire study population, the prevalence of valve disease in Chambers et al.’s study increased to 15%, according to our data, where we observed an increased prevalence of valve disease to 45.1% [46]. According to the oxVALVE PCS study, the prevalence of moderate or severe undiagnosed valve diseases in 65 years or older patients is 11.9%; this data differs from our results, which showed a prevalence of patients with undiagnosed valve disease of about 45.1% [14]. In the oxVALVE PCS study, the prevalence of undiagnosed aortic stenosis is 1.3%. This resembles the data of our study, where the prevalence of undiagnosed aortic stenosis was 3.4% [14]. Aortic stenosis and valve disease in general, like other pathologies, define specific and appropriate anesthetic management [47,48,49]. Not all valvular pathologies are clinically significant; mild mitral or tricuspid valve diseases need only a change in lifestyle, according to the most recent guidelines [28,39]. Patients with moderate–severe tricuspid insufficiency had a greater risk of mortality and morbidity 1 month after surgery, not correlated with RCRI scores or ejection fraction [50]. Moreover, severe aortic stenosis is associated with a higher risk of MACEs at 1 month after surgery (18.8%), whether symptomatic or not; the risk of mortality due to MACE was similar if correlated with class I or II of RCRI score and increased only with an RCRI score > 2 [51]. The importance of a pEco-s was supported by the diagnosis of the moderate–severe and severe diseases in our population; indeed, the finding led to a change in anesthesia by using CSA. CSA allows better control of analgesia with optimal hemodynamic stability and low variance in blood pressure [52]; better hemodynamic stability of CSA could explain why the evaluated patients reported no incidence of MACE, angina, or ICU admission.

Echocardiography is the cardiac imaging with the highest sustainability, the lowest CO_2_ emission and environmental footprint, no ionizing radiation, and the lowest cost compared to other cardiac imaging, such as scintigraphy and PET cardiography [53]. Even though the assessment of valvopathy is accurate, the assessment of left ventricular function may not be based on a single evaluation due to the variability of the operators, the estimation method, and the patient’s therapy [54,55]. According to the 2022 ESC guidelines on preoperative cardiac assessment in NCS, the use of echocardiography should be considered in all patients aged more than 70 years old who have poor functional capacity; furthermore, It is also recommended to adjust the risk for all patients who cannot climb two flights of stairs and are undergoing intermediate-risk NCS, such as orthopedic surgery [8,9,28,29]. According to Bossone et al., a prescreen with RCSI seems to be more useful and will be needed for echocardiography screening in cases where the score is more than 1 [56].

Finally, this study seems to confirm the doubts about the use of METs and ACS-NSQIP tools for cardiac risk assessment due to possible confounding factors, such as the functional limitations caused by orthopedic disease. However, only RCRI seemed to distinguish mild, moderate, or severe MACE risk in elderly patients undergoing major orthopedic surgery. The use of echocardiography could be an important instrument for the evaluation of this elderly patient and could discover pathologies at high risk of complications. Despite these findings, it is not possible to clearly establish the clinical impact of pEco-s for moderate disease and if targeted anesthesia management based on pEco-s findings can reduce the MACE. More studies are needed to clarify the cost–benefit of pEco-s.

It is important to note that this study has some limitations. First, we need to consider potential biases due to the retrospective nature of the study. Second, the study has a limited sample size, and it includes a specific type of patients undergoing orthopedic hip and knee replacement revision surgery. Third, the study used a limited number of risk scores. Fourth, echocardiographic screening was not consistently performed by the same operator, and incomplete data were retrieved (left atrial anteroposterior dimension, interventricular septal wall thickness, left ventricular (LV) posterior wall thickness, LV end-diastolic diameter, LV end-systolic diameter tricuspid or mitral anulus plane excursion, and the trans-valvular gradients). These limitations highlight the need for further analysis of larger groups of patients to validate our findings.

## 5. Conclusions

Our study found that performing a preoperative echocardiography in patients with functional limitations can reveal misrecognized valvopathies, most of which are mild. However, a small percentage of new valvopathies were moderate–severe and required modification for anesthesiologic management tailored to the patient. Indeed, the finding of severe disease changed the most frequent approach of single-shot spinal anesthesia for a CSA with a lower hemodynamic impact. It is important to note that preoperative evaluation by echocardiography can be expensive and requires significant human resources.

The scarce number of patients is a limitation of our study. A multicenter randomized trial for elderly patients with functional limitations would be necessary to evaluate the adequacy of echocardiography as preoperative screening.

## Figures and Tables

**Table 1:** Sample characteristics and preoperative parameters.

*N* = 59	Descriptive Statistic
**Characteristic**	**Mean ± SD**
Age (years)	72.5 ± 6.9
Weight (kg)	81.3 ± 14.9
Height (cm)	164.0 ± 7.6
BMI (kg/m^2^)	30.5 ± 5.1
	
**Sex**	***N* (%)**
Male	15 (25.4%)
Female	44 (74.6%)
	
**Type of surgery**	
Hip revision surgery	29 (49.1%)
Knee revision surgery	30 (50.9%)
	
**Preoperative assessment**	**Mean ± SD**
eGFR	76.7 ± 19.7
Basal Hb (g/dL)	12.6 ± 1.7
Systolic BP (mmHg)	135.4 ± 15.5
Diastolic BP (mmHg)	78.5 ± 9.0
Mean BP (mmHg)	98.3 ± 15.1
	
**Functional status evaluation**	
KOOS-I (daily function)	16.2 ± 5.1
KOOS-I (sport function)	8.3 ± 4.7
HOOS (daily function)	15.6 ± 6.0
HOOS (sports function)	8.1 ± 5.1
	
**ASA physical status**	***N* (%)**
I	3 (5.1%)
II	35 (59.3%)
III	17 (28.8%)
IV	4 (6.8%)

eGFR (estimated Glomerular Filtration Rate); BP (Blood Pressure); Hb (Hemoglobin); KOOS-I (Knee Injury and Osteoarthritis Outcome Score-Italian preoperative version); HOOS (Hip Osteoarthritis Outcome Score-Italian Version).

**Table 2:** Descriptive statistics of cardiac risk factors.

Risk Factors	Prevalence *N* (%)
**Obesity**	28 (45.9%)
BMI 30–34.99 kg/m^2^	19 (67.9%) ^†^
BMI 35–39.99 kg/m^2^	6 (21.4%) ^†^
BMI ≥ 40 kg/m^2^	3 (10.7%) ^†^
	
**Smoke**	12 (19.7%)
	
**Cardiovascular Disease**	55 (90.1%)
Hypertension	51 (83.6%)
iLVEF	4 (6.5%)
Valvopathy	29 (47.3%)
Previous AMI	7 (11.5%)
Aortic aneurysm	3 (4.9%)
	
**Other Disease**	
Diabetes	12 (20.3%)
COPD	20 (32.8%)
OSAS	1 (1.6%)
Rheumatologic disease	6 (9.8%)

iLVEF (Impaired Left Ventricular Ejection Fraction); AMI (Acute Myocardial Ischemia); COPD (Chronic Obstructive Pulmonary Disease); OSAS (Obstructive Sleep Apnea Syndrome). ^†^ The relative frequencies in percentual are referred to the obese patients (*N* = 28).

**Table 3:** Cardiac risk scores analysis before and after recrudescence of orthopedic disease METs, preoperative RCSI, and preoperative ACS-NSQIP.

Score	Relative Frequencies of Patients with High-Risk
**METs < 4 ^¶^**	***N* (%)**
Prior orthopedic disease	7 (11.9%)
After orthopedic disease	54 (91.5%)
	
**RCRI**	***N* (%)**
Class I	41 (69.5%)
Class II	17 (28.8%)
Class III	1 (1.7%)
Class IV	0 (0.0%)
	
**ACS-NSQIP**	***N* (%)**
High-risk score (>0.006) in hip revision patient	29 (100%) *
High-risk score (>0.006) in knee revision patient	24 (80.0%) ^§^

^¶^ The χ^2^ test between the distribution before and after surgery showed a *p* < 0.001; the χ^2^ test between the hip and knee surgery showed a *p* = 0.07. * The mean ± SD of the score is 0.011 ± 0.005. ^§^ The mean ± SD of the score is 0.013 ± 0.012. METs (Metabolic Equivalents Estimated); RCRI (Revised Cardiac Risk Index); ACS-NSQIP (American College of Surgeons—National Surgical Quality Improvement Program).

**Table 4:** Analysis of prevalences of impaired left-ventricular ejection fraction and valvopathy before and after echocardiography screening.

Disease	Pre-Echocardiography Known Pathology *N* (%) *	Post-Echocardiography Diagnosed Disease *N* (%) *	Difference between Pre- and Post-Echocardiography *N*	χ^2^	*p*-Value
**iLVEF and Valvopathy**	28 (45.9%)	47 (77.0%)	19	8.3	<0.001
**iLVEF**	4 (6.55)	7 (11.5%)	3	<0.001	1
**Patients with one or more valvopathy**	11 (18.6%)	23 (45.1%)	12	4.5	0.03
**Total Valvopathy**	27	43	25	12.0	<0.001
**Aortic valve**	11 (18.0%)	21 (34.4%)	10	<0.001	1
**Pulmonary Valve**	1 (1.7%)	1 (1.7%)	0	<0.001	1
**Tricuspid Valve**	18 (29.5%)	25 (41.0%)	7	<0.001	1
**Mitral Valve**	23 (37.7%)	35 (57.4%)	8	0.36	0.55

iLVEF (impaired Left Ventricular Ejection Fraction). * The frequencies are referred to as the total sample size (*N* = 59).

**Table 5:** Descriptive statistics (absolute and relative frequencies) of all grade valvopathy distribution among the newly diagnosed valve diseases, distribution of the chosen anesthetic management, and postoperative MACE.

Disease (Difference Pre- and Post-Echocardiography)	Grade	Absolute and Relative Frequency N (%) Referred to Newly Diagnosed Pathology
**Aortic valve (10)**	° Mild	7 (70.0%)
	° Moderate	1 (10.0%)
	Moderate–Severe	0 (0.0%)
	* Severe	2 (20.0%)
**Tricuspid valve (7)**	°Mild	6 (85.7%)
	Moderate	0 (0.00%)
	° Moderate–Severe	1 (14.3%)
	Severe	0 (100%)
**Mitral valve (8)**	° Mild	8 (100%)
	Moderate	0 (0.0%)
	Moderate–Severe	0 (0.0%)
	Severe	0 (0.0%)
**Total (25)**	° Mild	21 (84.0%)
	° Moderate	1 (4.0%)
	° Moderate–Severe	1 (4.0%)
	Severe	2 (8.0%)
		
**Anesthesia procedure**	Epidural	0 (0.0%)
	Subarachnoid	56 (95.0%)
	^ʄ^ CSA	3 (5.0%)
		
**Major cardiac events reported after surgery (0)**	**Symptomatic MI**	0 (0.0%)
	Known cardiac disease	0 (0.0%)
	Newly diagnosed disease	0 (0.0%)
	**Angina pectoris (stable or unstable**)	0 (0.0%)
	Known cardiac disease	0 (0.0%)
	Newly diagnosed disease	0 (0.0%)
	**ICU admission**	0 (0.0%)
	Known cardiac disease	0 (0.0%)
	Newly diagnosed disease	0 (0.0%)
	**Postoperative death**	0 (0.0%)
	Known cardiac disease	0 (0.0%)
	Newly diagnosed disease	0 (0.0%)
	**Total events**	0 (0.0%)

° Insufficiency; * stenosis; ^ʄ^ CSA was performed in the patients affected by moderate–severe and severe valvopathy; the χ^2^ test showed a *p* = 0.002. It was not possible to run a χ^2^ test because the dichotomous data were practically linear. ICU, Intensive Care Unit; CSA, Continuous Spinal Anesthesia.

## Data Availability

No new data were created or analyzed in this study. Data sharing is not applicable to this article.
